# Comprehensive Characterization of Acorns from Selected *Quercus* Species: Nutritional Composition, Phytochemical Profile and Potential Relevance to Swine Nutrition

**DOI:** 10.3390/molecules31172967

**Published:** 2026-08-25

**Authors:** Anna Zwyrzykowska-Wodzińska, Przemysław Bąbelewski, Anna Marietta Salejda, Bogdan Jarosz, Maciej Włodarczyk, Aleksandra Szmaja, Joanna Miedzianka, Marta Czaplicka, Patryk Jagoda, Zdenek Zadák, Anna Jankowska-Mąkosa, Damian Knecht

**Affiliations:** 1 Institute of Animal Breeding, Wroclaw University of Environmental and Life Sciences, 51-630 Wrocław, Poland; anna.jankowska-makosa@upwr.edu.pl (A.J.-M.); damian.knecht@upwr.edu.pl (D.K.); 2 Department of Horticulture, Wroclaw University of Environmental and Life Sciences, 50-363 Wrocław, Poland; przemyslaw.babelewski@upwr.edu.pl (P.B.); marta.czaplicka@upwr.edu.pl (M.C.); patryk.jagoda@upwr.edu.pl (P.J.); 3 Faculty of Biotechnology and Food Science, Wroclaw University of Environmental and Life Sciences, 51-630 Wrocław, Poland; anna.salejda@upwr.edu.pl (A.M.S.); joanna.miedzianka@upwr.edu.pl (J.M.); 4 Department of Food Chemistry and Biocatalysis, Wroclaw University of Environmental and Life Sciences, 50-375 Wrocław, Poland; bogdan.jarosz@upwr.edu.pl; 5 Department of Pharmacognosy and Herbal Medicines, Wroclaw Medical University, Borowska 211A, 50-556 Wrocław, Poland; maciej.wlodarczyk@umw.edu.pl; 6 Department of Clinical Biochemistry and Diagnostics, Faculty of Medicine and University Hospital, Charles University, Sokolska Str. 581, 500 05 Hradec Kralove, Czech Republic; zdenek.zadak@fnhk.cz

**Keywords:** acorns, pig nutrition, chemical composition

## Abstract

Acorns represent a traditional seasonal feed resource for pigs in Mediterranean production systems; however, comparative information on the nutritional composition and safety of acorns from Central European oak species remains scarce. The present study comprehensively characterized acorns of *Quercus robur*, *Q. petraea*, and *Q. rubra* to assess their potential relevance to pig nutrition based on their chemical composition. Proximate composition, mineral content, fatty acid and amino acid profiles, phenolic compounds, tannins, triterpenoid acids, and heavy metal concentrations were determined using analytical methods. Differences among the analyzed acorn samples were observed in nutritional composition and phytochemical profile. Acorns from all three species were characterized by a high proportion of unsaturated fatty acids, particularly oleic acid, a favourable amino acid profile, and the presence of phenolic compounds, tannins, and triterpenoid acids. Heavy metal concentrations remained below the maximum levels established by European legislation for feed materials, indicating that the analyzed acorns showed no heavy-metal-related exceedances of the applicable European limits for feed materials. The results indicate that acorns from Central European oak species represent a locally available feed resource of potential relevance to pig nutrition. This comprehensive chemical characterization provides a scientific basis for future nutritional studies and further evaluation of acorns as sustainable feed materials.

## 1. Introduction

The genus *Quercus* belongs to the family *Fagaceae*, which also includes trees of the genera *Fagus* and *Castanea*. The fruits of these trees are edible, single-seeded nuts that are enclosed in a woody seed coat of varying shape. The genus *Quercus* is rich in numerous species, the vast majority of which grow in the Northern Hemisphere, while only a few are found in the Southern Hemisphere. It is estimated that there are about 400 different species worldwide, and 30 species in Europe alone. In Poland, two species grow in natural stands in deciduous and mixed forests. *Q. robur*, occurs throughout Europe (except for the northern and southernmost regions) and in southwestern Asia. The second European species is *Q. petraea*, whose northeastern range of natural occurrence ends in Poland. These two species are highly valuable forest trees growing in nutrient-rich habitats and are also popular in landscaping, where they are commonly planted in parks, residential green spaces, large public squares, and as street trees. These oaks grow into massive, long-lived trees reaching ages of several hundred years, and individual trees can live for over 1000 years. They produce acorns practically every year, which are a favourite food of wild mammals such as wild boars, dormice, and flying squirrels, as well as birds such as jays, capercaillies, and grosbeaks.

Another, controversial species found in Poland is *Q. pubescens*, which grows at only one site in Bielinek on the Oder River, a nature reserve. It is believed that this stand was established with human intervention, as it is located in the north, over 1000 km outside the natural range of this species in Europe, possibly as the result of domestic animal feeding during long distance transporting [1].

Another species cultivated in Poland is *Q. rubra*, which occurs naturally in eastern North America—from southeastern Canada to the eastern states of the U.S. It belongs to section *Lobatae* (red oaks), whereas the native European species *Q. robur* and *Q. petraea* belong to section *Quercus* (white oaks). Acorns of *Q. rubra* mature in the second year after flowering. The species was introduced to Poland in the 19th century as an ornamental tree due to its attractive red autumn foliage. It is a very popular ornamental tree in parks, large green spaces, and along streets. It is considered a species with lower soil requirements than local oak species. When planted in forests, it has proven to be an invasive species; it also self-seeds in synanthropic sites in cities and is currently a nuisance to natural forest ecosystems [2].

Among the numerous ecosystem services provided by oaks, acorns have played a particularly important role as a traditional source of nutrients for both humans and animals.

Oak fruits (acorns) were used as carbohydrate-rich human food by Native Americans (*Q. lobata* Née and other species) after proper preparation [3,4]. Similarly, in Europe people utilized acorns from *Q. robur* L. (syn. *Q. pedunculata* Hoffm.) and *Q. petraea* (Matt.) Liebl. [5], *Q. ilex* L. [6] and *Q. infectoria* G. Olivier [7]. Precise modern descriptions of the yielding, collection, and preparation of acorns are also available [8,9].

Acorns have been used in animal feeding for centuries, particularly in Mediterranean countries where oak woodlands are an integral part of traditional livestock production. In these systems, naturally fallen acorns constitute a valuable seasonal feed resource for grazing animals during autumn. Although their use is primarily associated with pig production, acorns have also been utilized in the feeding of other livestock species [10,11].

Interest in acorns has increased in recent years as livestock production seeks alternative feed resources that can partially replace conventional cereals. Acorns are primarily a source of carbohydrates, but they also contain lipids, minerals and a variety of secondary metabolites, including tannins, phenolic compounds and triterpenoids [11,12,13]. The concentrations of these compounds differ among *Quercus* species and are influenced by environmental conditions, geographical origin and fruit maturity. As a result, the nutritional value of acorns may vary considerably between studies.

Feeding trials indicate that acorns can be incorporated into the diets of several livestock species without adverse effects on animal performance when appropriate inclusion levels are used. In lambs, partial replacement of barley with acorns maintained growth performance, nutrient digestibility and carcass characteristics [14]. Comparable results have been reported in rabbits, where dietary acorns improved the fatty acid composition of adipose tissue [15]. In broiler chickens, diets containing green oak acorns improved antioxidant status and selected health parameters [16].

Among farm animals, pigs are the species most frequently associated with acorn feeding. In Mediterranean production systems, acorns are consumed during the finishing period and contribute to the characteristic quality of pork. Experimental studies have also shown that acorns may influence nutrient digestibility, fecal characteristics [17], and the fatty acid composition of meat, particularly by increasing the proportion of monounsaturated fatty acids [18].

The use of acorns in animal nutrition is nevertheless limited by their variable chemical composition and relatively high tannin content, both of which influence their nutritional value. These factors should be considered when formulating diets and comparing results obtained in different studies. Therefore, detailed characterization of the chemical composition of acorns is necessary to evaluate their potential as feed ingredients and to explain the differences reported in animal feeding experiments.

The use of acorns in animal nutrition is constrained by their high content of tannins and other bioactive compounds, whose effects depend on their concentration, animal species and dietary inclusion level. We understand that consumption of tannin-rich food can lead to numerous effects—from mild specific astringent and bitter taste, through GIT (gastrointestinal tract) sensation, to the serious damage to parenchymal organs involved in detoxifying the body (liver, kidneys), oaks are typical source of tannins [17,19,20]. However, to our best knowledge there is no scientific report concerning pig intoxications resulting from acorns during pannage (fr. glandée, ger. Eichelmast, span. montanera, port. montado) while such data exists for other livestock like cattle or horses [21,22]. This apparent tolerance may be explained, at least in part, by physiological adaptations of pigs to tannin-rich diets, including increased production of salivary proline-rich proteins that bind tannins and reduce their biological activity [23]. The general opinion received from the pig farmers is that acorns should be mature, prolonged shelf life is beneficial when properly stored, if only they remain dry and unmoldy, which generally corresponds with the knowledge on the time- and redox-instability of tannins.

The effect of tannins and polyphenols on the health of farm animals is a matter of debate [24,25]. On the one hand, tannins, by binding to the mucous membranes of the GIT, reduce its permeability (including to nutrients). They can also bind to nutrient molecules, slowing or preventing their absorption through this pathway. Furthermore, by binding to digestive enzymes in the lumen of the GIT, they hinder the breakdown of food into absorbable components. Their effect on the gastrointestinal flora is also observable. Finally, simple phenols resulting from their breakdown by microflora can be absorbed from the GIT and affect the liver or kidneys [19,20,26,27,28,29].

On the other hand, polyphenols generally are noticed for their antioxidant and free-radical-scavenging effects, even if they are thought to act only in the GIT lumen. Additionally, there are reports on their favourable influence against ferroptosis-related damages of GIT [30,31]. However, the applied commercial polyphenol-rich extract was not characterized chemically nor was its origin disclosed, which makes the interpretation of experiment results doubtful.

In other work, a commercial product based on sweet chestnut tannins was proven to help with gastric illnesses in livestock [32,33].

Regarding the other small-molecule acorn constituents, we do not have much recent data on triterpenes and their effects on livestock condition and growth. Generally, there are reports that the use of extracts containing such compounds may be associated with GIT disturbances [19], but a distinction should be made between studies on the effects of saponins (amphiphilic terpenoid glycosides) and free triterpenoid acids.

There are reports indicating potential antinutritional effects of triterpenoid acids [34]. However, due to their low visibility in common UV detectors (compared with, e.g., phenolics), we expect TTP (triterpenes, particularly triterpenoid acids) to be routinely overlooked and remain unquantified or unreported in experimental animal foods.

The aim of this study was to comprehensively characterize acorns from selected *Quercus* species by determining their nutritional composition, amino acid and fatty acid profiles, mineral composition, heavy metal content and phytochemical fingerprint, in order to assess their potential relevance to swine nutrition based on their nutritional and phytochemical composition.

## 2. Results

### 2.1. Proximate Composition of Acorns

The proximate composition of the pre-dried, fresh-like acorn material from the three investigated *Quercus* species is presented in Table 1. On an as-is basis, dry matter content ranged from 57.16% in *Q. petraea* to 67.49% in *Q. rubra*, while *Q. robur* contained 65.37% dry matter. Crude ash content was comparable among the analyzed species, ranging from 1.24% in *Q. petraea* to 1.28% in *Q. rubra*. Similarly, crude protein content showed little variation, ranging from 3.20% in *Q. petraea* to 3.27% in *Q. rubra*.

In contrast, greater variation was observed in crude fat and crude fibre contents. On an as-is basis, *Q. rubra* was characterized by the highest crude fat content (9.04%), whereas substantially lower values were recorded for *Q. robur* (2.62%) and *Q. petraea* (2.10%). A similar pattern was observed for crude fibre, with *Q. rubra* containing 16.63%, compared with 5.76% and 5.73% in *Q. robur* and *Q. petraea*, respectively. Overall, *Q. rubra* showed the highest crude fat and crude fibre contents, whereas *Q. robur* and *Q. petraea* showed lower values for these components.

### 2.2. Amino Acid Composition of Acorns

The amino acid composition of acorns from the three investigated *Quercus* species is presented in Table 2. A total of 17 proteinogenic amino acids were identified.

The total amino acid content varied among the analyzed species, with the highest value recorded in *Q. petraea* (36.53 mg/g), followed by *Q. robur* (31.50 mg/g) and *Q. rubra* (29.77 mg/g).

Glutamic acid and aspartic acid were the predominant amino acids in all analyzed acorns. The highest concentrations of both amino acids were determined in *Q. petraea*, whereas the lowest glutamic acid content was observed in *Q. robur* and the lowest aspartic acid concentration in *Q. rubra*.

Among the essential amino acids, leucine, arginine, lysine, valine and threonine occurred at the highest concentrations. In contrast, methionine and cysteine were present at the lowest levels in all analyzed species. *Q. petraea* exhibited the highest concentrations of most analyzed amino acids, whereas *Q. rubra* generally contained the lowest values.

### 2.3. Fatty Acid Composition of Acorns

The fatty acid composition of acorns from the three investigated *Quercus* species is presented in Table 3. Oleic acid (C18:1 cis-9) was the predominant fatty acid in all analyzed samples, accounting for 48.11–59.73% of total fatty acids. The highest proportion was observed in *Q. rubra*, whereas *Q. robur* and *Q. petraea* exhibited similar values (48.11% and 48.56%, respectively).

Linoleic acid (C18:2 cis-9,12) represented the second most abundant fatty acid, ranging from 27.46% in *Q. rubra* to 32.08% in *Q. robur.* Palmitic acid (C16:0) was the predominant saturated fatty acid and varied between 9.01% in *Q. rubra* and 13.52% in *Q. robur*.

Smaller amounts of α-linolenic acid (C18:3 cis-9,12,15), stearic acid (C18:0), arachidic acid (C20:0) and lignoceric acid (C24:0) were detected in all analyzed species showed a higher proportion.

Overall, *Q. rubra* was characterized by a markedly higher proportion of monounsaturated fatty acids, whereas *Q. robur* and *Q. petraea* contained relatively higher proportions of saturated and polyunsaturated fatty acids.

### 2.4. Ultra-High-Performance Liquid Chromatography–Quadrupole Time-of-Flight High-Resolution Mass Spectrometry (UHPLC-qTOF-HRMS) Fingerprinting

The data obtained with LCMS were compiled in Table 4, and supplemented by Figures S1 and S2.

Among hydrolyzable phenolics, 29 compounds were putatively classified as hydrolyzable tannins *sensu lato* (including simple phenolic glycosides, e.g., of ellagic acid) (Table 4). Among them, ellagic acid hexoside and pentoside (463, 10.11 min and 433, 10.97 min, respectively) were typical for *Q. petraea* and *Q. robur,* while two tannins ([M−H]^−2^ at 783 *m*/*z*, 8.94 min, [M−H]^−2^ at 860 *m*/*z*, 11.97 min) together with presumable dominant cretanin were typical for *Q. rubra*.

Additionally, we observed eight other phenolics: dominant ellagic acid in *Q. petraea*; comparable intensities of gallic and ellagic acids in *Q. robur*; and, in *Q. rubra*, dominant free gallic acid and methyl gallate, accompanied by a putative *p*-methyl gallic alcohol (169.05, 5.38 min).

Referring to the condensed tannins, we have not detected any of the simple catechins, dimeric, trimeric, or tetrameric procyanidins in significant amounts. Concerning the previous work [35], this may be a result of another detector type, other specific parameters of detection, or a different composition of plant material.

In this study, a significant number of similar substances were detected and tentatively identified as terpenoids (36 simple triterpenoids, 11 triterpenoid hexosides/hexosyl esters, and 2 triterpenoid gallates). These were specific mostly to *Q. petraea* acorns and were probably not reported previously from acorns of *Q. petraea*, *Q. robur,* and *Q. rubra*. In the naming given in Table 4, when a triterpene compound formed a significant adduct with formic acid (from the mobile phase), it was marked by providing two anions differing by 46 Da (e.g., 565/519, 15.92 min), with the first being more intense than the second.

Three of the presumed simple triterpenoids, with pseudomolecular anions at 503 *m*/*z*, eluting at 17.15, 17.25, and 18.41 min—provisionally named as arjungenin isomers—were dominant in this group. Two other ones possessed formulas corresponding to compounds isolated previously from *Q. serrata* acorns (519, 15.92 min and 519, 16.04 min) [36]. Two presumed gallates could be referred to as those isolated from *Q. petraea* wood [37], but the chemical standards are not available.

Among the other detected compounds, nine different jasmonates were tentatively identified, including presumed sulfates of tuberonic (305, 9.38 min; all samples) and dihydrotuberonic acid (307, 9.67 min), five isomers of tuberonic acid hexoside ([M−H]^−^ at 387 *m*/*z*, at 9.09, 9.27, 9.38, 9.53, 9.61, and 10.07 min) and two isomers of tuberonic acid ([M−H]^−^ at 225 *m*/*z*, at 10.24 and 10.35 min). These compounds are classified as plant hormones and can play a role during the development of new plants from seeds [38].

Some compounds remain only detected but not defined more precisely after Chemical Abstracts screening.

### 2.5. Macroelement, Microelement, Heavy Metal and Nitrate Contents

The contents of macroelements, microelements, heavy metals and nitrates in acorns of the investigated *Quercus* species are presented in Table 5.

Among the analyzed macroelements, phosphorus content did not differ significantly among the investigated species, although the highest concentration was recorded in *Q. petraea* (106.0 mg/kg fresh-like matter) and the lowest in *Q. robur* (92.3 mg/kg fresh-like matter). In contrast, calcium, potassium, magnesium and sodium contents differed significantly between the analyzed species. Calcium concentration ranged from 83.2 mg/kg fresh-like matter in *Q. robur* to 133.9 mg/kg fresh-like matter in *Q. rubra*. The highest potassium (700.5 mg/kg fresh-like matter) and magnesium (44.6 mg/kg fresh-like matter) contents were determined in *Q. petraea*, whereas the lowest concentrations of both elements were found in Q. rubra (539.5 and 27.1 mg/kg fresh-like matter, respectively). Sodium content ranged from 16.2 mg/kg fresh-like matter in *Q. petraea* to 21.4 mg/kg fresh-like matter in *Q. rubra*. Among the analyzed microelements, zinc, iron, copper and molybdenum contents did not differ significantly among the investigated species. Boron concentration was significantly higher in *Q. rubra*, whereas *Q. petraea* and *Q. robur* showed comparable values. Manganese content differed significantly among all three *Quercus* species.

Cadmium concentration was comparable in *Q. rubra* and *Q. robur*, whereas *Q. petraea* differed significantly from both species. Significant differences were also observed for strontium and aluminum contents, while lead concentration remained similar among the analyzed acorns.

Nitrate content differed significantly among the investigated *Quercus* species. The highest concentration was recorded in *Q. petraea,* whereas the lowest was observed in *Q. rubra*.

## 3. Discussion

The growing interest in sustainable and locally available feed resources has renewed attention to underutilized plant materials that may partially replace conventional feed ingredients in animal nutrition. Among these, acorns have received increasing scientific attention because of their favourable chemical composition and their potential relevance as an alternative feed resource. Their nutritional value depends on the balance between desirable nutrients and compounds that may limit nutrient utilization. Although acorns have been investigated as feed ingredients for several farm animal species, their traditional use is particularly associated with pig production, where acorn consumption has also been linked to the quality of pork and traditional meat products. Rather than being considered a direct replacement for conventional feed ingredients in intensive production systems, acorns may be viewed as a locally available seasonal feed resource or as a potential dietary component, particularly where oak resources are readily available. Their relevance to pig production has been documented especially in free-range and semi-extensive systems, in which pigs naturally consume acorns during the autumn season and where this traditional feeding practice has been associated with changes in the nutritional and sensory characteristics of pork products. Against this background, the present study aimed to comprehensively characterize the nutritional and phytochemical composition of acorns from selected *Quercus* species, including proximate composition, mineral content, fatty acid and amino acid profiles, and the occurrence of bioactive phytochemicals, and to discuss their potential relevance to pig nutrition based on the obtained compositional data [12,39,40,41,42].

The proximate composition of acorns confirmed considerable variation among the investigated *Quercus* species, which is consistent with previous studies demonstrating that the chemical composition of acorns is influenced by species, geographical origin, climatic conditions, soil characteristics and fruit maturity [12,39,43,44]. Özcan et al. (2012) [43] additionally suggested that differences in the chemical composition of acorns may result from both species-specific characteristics and environmental conditions.

However, because all acorns analyzed in the present study were collected from the same geographical location under comparable environmental conditions, major differences related to geographical origin were minimized. Nevertheless, the use of one composite sample per species does not allow species-specific effects to be separated from variation among individual trees or microsite conditions. Therefore, the observed differences should be considered descriptive and require confirmation using independently collected samples from multiple trees and locations.

The analyzed acorns exhibited the compositional characteristics typical of the genus *Quercus,* namely a carbohydrate-rich composition, moderate crude fat content and relatively low crude protein concentration. Acorns are generally regarded as an energy-rich raw material owing to their high starch content (47–60%) and moderate oil content (7–14.4%), although considerable interspecific variation has been reported [39,40,45].

Among the investigated species, *Q. rubra* was characterized by the highest crude fat (9.04%) and crude fibre (16.63%) contents, whereas *Q. robur* and *Q. petraea* contained considerably lower crude fat (2.62% and 2.10%, respectively) and crude fibre (5.76% and 5.73%, respectively). Crude protein and ash contents remained relatively similar among all three species. The crude fat content of *Q. rubra* observed in the present study exceeded that of the remaining species, confirming that crude fat differs markedly among oak species even when acorns are collected under identical environmental conditions. A similar tendency was reported by Özcan (2008) [45], who determined an oil content of approximately 7.4% (dry matter) in *Q. rubra* acorns collected in Turkey, whereas Vinha et al. (2016) [39] likewise described substantial interspecific variation in crude fat content among European *Quercus* species. Although direct numerical comparisons should be interpreted with caution because most published values are expressed on a dry matter basis—whereas the present results are presented on a fresh weight basis—the overall compositional trends remain comparable.

The mineral composition of acorns varied considerably among the investigated *Quercus* species, confirming that oak fruits represent an important source of essential macroelements. Similar observations have been reported previously, indicating that both species and environmental conditions influence mineral accumulation in acorns [39,43,46]. However, because all acorns analyzed in the present study were collected from the same geographical location under comparable environmental conditions, the observed differences may reflect variation among *Quercus* species, although the contribution of variation among individual trees or microsite conditions cannot be excluded.

Potassium was the predominant macroelement in all analyzed acorns, which is consistent with numerous previous studies identifying potassium as the major mineral constituent of *Quercus* fruits [43,47]. In the present study, the highest potassium concentration was determined in *Q. petraea*, followed by *Q. robur* and *Q. rubra*. A similar predominance of potassium was reported for *Q. suber*, *Q. ilex* and several Turkish *Quercus* species, although absolute concentrations differed depending on species and geographical origin.

Among the remaining macroelements, *Q. petraea* was characterized by the highest phosphorus and magnesium concentrations, whereas *Q. rubra* accumulated the greatest amount of calcium. Özcan et al. (2012) [43] likewise reported pronounced variation in calcium, phosphorus and magnesium contents among *Quercus* species, suggesting that mineral accumulation is largely genetically determined. The consistency of these observations with the present study is particularly noteworthy because all analyzed acorns originated from the same collection site. Consequently, the influence of environmental variability was minimized, allowing species-related differences in mineral accumulation to be interpreted with greater confidence.

Overall, the obtained results confirm that each oak species possesses a characteristic mineral profile. Therefore, mineral composition should be considered together with proximate composition, fatty acid profile and amino acid composition when evaluating the overall nutritional value of acorns.

The concentrations of heavy metals determined in the analyzed acorns were generally low, indicating that the investigated material does not pose a potential risk from the perspective of feed safety. Similar findings have been reported for several *Quercus* species, where heavy metal concentrations remained within acceptable limits despite considerable variation among species and collection sites [43,46]. Özcan et al. (2012) [43] suggested that differences in heavy metal accumulation may be associated with variation among *Quercus* species, soil properties and local environmental conditions, emphasizing the importance of collection site selection.

The concentrations of all analyzed heavy metals were below the maximum levels established by Directive 2002/32/EC of the European Parliament and of the Council on undesirable substances in animal feed [48], indicating that the analyzed acorns comply with current European feed safety requirements. These findings indicate that acorns collected from appropriately selected locations may warrant further evaluation as a potential feed material, provided that their elemental composition is routinely monitored.

From a practical perspective, the utilization of acorns in pig nutrition should be considered within the existing biosecurity framework applied to feed materials intended for pigs. Although acorns have traditionally been used as a feed resource for various livestock species, their use in pig production requires compliance with biosecurity measures aimed at preventing African swine fever (ASF), including minimizing the risk of contact with wild boar and environmental contamination, in accordance with Commission Implementing Regulation (EU) 2023/594 [49]. Importantly, these considerations are not unique to acorns but also apply to other feed materials harvested from agricultural or natural environments that may be accessible to wild boar. Appropriate storage or processing procedures, where required by national biosecurity regulations, may further reduce potential sanitary risks associated with feed materials collected from the environment. Consequently, managed green areas with negligible access of wild boar may represent one potential source of acorns, provided that local regulations permit their collection and appropriate quality and safety assessments are performed. Nevertheless, because the elemental composition of acorns is strongly influenced by local environmental conditions [43], acorns intended for pig feeding should also be evaluated for heavy metal contamination and verified for compliance with Directive 2002/32/EC [48] before incorporation into animal diets. Under the conditions of the present study, the low concentrations of heavy metals support the further evaluation of acorns collected from managed urban green areas as a potential feed material for pigs.

The fatty acid composition of the analyzed acorns was typical of the genus *Quercus*, with unsaturated fatty acids clearly predominating over saturated fatty acids. Similar profiles have been reported for numerous oak species, irrespective of their geographical origin, with oleic, linoleic and palmitic acids representing the major fatty acids of acorns [39,46,50,51].

Oleic acid (C18:1n-9) was the predominant fatty acid in all three investigated species, accounting for 48.11–59.73% of the total fatty acids. The highest proportion was observed in *Q. rubra* (59.73%), whereas *Q. petraea* and *Q. robur* contained comparable amounts (48.56% and 48.11%, respectively). These values fall within the range reported for other *Quercus* species, where oleic acid generally constitutes 42–67% of total fatty acids [46,50].

Linoleic acid (C18:2n-6) represented the second most abundant fatty acid, ranging from 27.46% in *Q. rubra* to 32.08% in *Q. robur.* Similar values have been reported for Mediterranean oak species, where linoleic acid usually accounts for 16–43% of total fatty acids [39,51]. Among saturated fatty acids, palmitic acid predominated, with concentrations ranging from 9.01% in *Q. rubra* to 13.52% in *Q. robur*, which is consistent with previous reports identifying palmitic acid as the major saturated fatty acid in acorns [46,50].

Although the overall fatty acid profiles were comparable, differences among the analyzed acorn samples were observed. The markedly higher oleic acid concentration in *Q. rubra* was accompanied by lower proportions of both palmitic and linoleic acids. In contrast, *Q. petraea* contained the highest concentration of α-linolenic acid (3.27%), which was almost nine-fold higher than that determined in *Q. rubra* (0.38%). Because all acorns analyzed in the present study were collected from the same geographical location under comparable environmental conditions, these differences may reflect variation among *Quercus* species, although the contribution of variation among individual trees or microsite conditions cannot be excluded.

The predominance of oleic acid observed in all analyzed acorns may be of particular relevance to pig nutrition. Acorn-based feeding has repeatedly been shown to increase the proportion of monounsaturated fatty acids in pork fat, thereby improving its oxidative stability and sensory quality, particularly in traditional Iberian production systems [50,52,53]. More recently, Belková et al. (2023) [42] demonstrated that the inclusion of 10% *Q. robur* acorns in diets for Přeštice Black-Pied pigs had no adverse effect on growth performance or carcass characteristics, providing literature-based evidence for the potential relevance of acorns as a dietary component under conventional indoor production conditions.

The total amino-acid content varied significantly among the analyzed acorns. The highest value was observed in acorns of *Q. petrea* (36.53 mg/g), followed by *Q.robur* (31.50 mg/g) and *Q.rubra* (29.77 mg/g). Similar variability among acorn species has been reported in previous studies, where differences in amino-acid composition were attributed to species, genotype, environmental conditions, and geographical origin [54].

In the present study, 17 amino acids were identified and glutamic acid and aspartic acid were the dominant amino acids. Glutamic acid content ranged from 5.39 to 6.12 mg/g, while aspartic acid ranged from 3.44 to 4.66 mg/g depending on the species. The highest content of both, glutamic and aspartic acid was found in the acorns of *Q. petrea*. Acorns obtained from *Q. robur* contained the least glutamic acid, and *Q. rubra* acorns contained the least aspartic acid. Similar patterns have been reported for acorns of different *Quercus* species, where glutamic and aspartic acids typically represent a large proportion of total amino acids in acorn proteins [39,51,54].

Among essential amino acids, relatively high levels of leucine, arginine, and valine were observed. Leucine ranged from 2.61 mg/g in *Q. rubra* to 3.08 mg/g in *Q. peterea*, while arginine ranged from 2.30 to 2.87 mg/g and valine from 1.72 to 2.08 mg/g. Comparable amino acid distributions were reported for acorns from several Turkish *Quercus* species, where leucine, lysine, and valine were identified as major essential amino acids [54]. Phenylalanine and isoleucine were also present at moderate levels in the analyzed samples (1.30–1.79 mg/g and 1.37–1.67 mg/g, respectively), which agrees with previously published amino-acid profiles of Mediterranean oak species [39].

In contrast, sulfur-containing amino acids occurred at much lower concentrations. Methionine ranged from 0.08 to 0.29 mg/g, while cysteine ranged from 0.09 to 0.17 mg/g. Low concentrations of methionine have also been reported in several studies on acorn proteins and are considered characteristic of many plant protein sources [39,55].

Beyond their nutritional value, the amino acid profile of acorns provides information relevant to their potential use in food and feed systems. Acorns have traditionally been used as animal feed, particularly for pigs and ruminants, where they serve as a natural source of energy and protein. Due to the imbalance in the provision of essential amino acids, mainly lysine, acorns are not recommended as the single feed available for the pig [56,57,58] but previous studies suggest that they may be used as a dietary component when appropriately balanced. Acorn-based diets have been shown to influence the nutritional quality and sensory characteristics of meat products, especially in traditional Iberian pig production systems [52]. In the study of Bělková et al. (2021) [59], experimental feed mixture containing 10% *Q. robur* acorns was balanced to the required level of amino acids and its use for the Přeštice black pied breed had no negative effect on the fattening parameters.

While the advantages of Mediterranean *Q. ilex* acorns are pointed out (relatively high content of fat and low of tannins), the pigs’ pannage on less fatty acorns was not extraordinary in Central and Western Europe [60,61,62]. Even economical estimations that 25 oak trees are needed to fatten one pig were given in the 19th c. [61]. However, traditional extensive pig production systems, particularly those involving seasonal use of forest resources, are becoming increasingly limited due to the implementation of biosecurity measures aimed at preventing the spread of transboundary infectious animal diseases, including African swine fever (ASF), classical swine fever (CSF) and foot-and-mouth disease (FMD) [49,63]. It is therefore essential to properly understand and select feed that affects meat quality in order to achieve, even a semblance of the quality of products from the past, through intensive farming.

Some comparative studies of the tannin content in Central European acorns have already been conducted [64,65], and numerous studies have been reviewed in recent publications [11,12]. Using a pharmacopoeial method, the authors showed that fruits of *Q. petraea*, *Q. robur*, and *Q. rubra* were generally similar from a chemical standpoint, with some sample variability noted [65]. However, during our research, we found that (1) pharmacopoeial hide powder, which is essential for conducting this experiment, seems too expensive to be used in routine testing, and (2) the tannins in acorns of native Central European oaks were not defined precisely. Thus, the aim of this study was to comparatively fingerprint the extracts from acorns of three species abundant in the region of Poland (*Q. petraea*, *Q. robur*, and *Q. rubra*) with UHPLC-MS and –MS2 to contribute to further research on this economically significant issue in pig farming.

During our LCMS screening we recorded over 100 compounds in a total in a typical 70% aqueous methanol extracts. As a novelty, we report numerous tentatively identified triterpenes and their glycosides or glycoesters, which are particularly abundant and variable in *Q. petraea* extract.

*Q. rubra* acorns tannins were defined previously with convincing UHPLC-q-orbitrap-MS and NMR confirmation [35]. *Q. petraea* and *Q. rubra* acorn tannins were also compared with HPLC [66]. Here, we confirm their results with a comparable composition of *Q. rubra* acorns.

When comparing tannin content in acorns with the Folin-Ciocalteau method, it was demonstrated that in all cases total phenolics amount of *Q. robur* was about two times higher than of *Q. ilex* [67], and the total phenolics amount for *Q. petraea*, *Q. robur*, and *Q. rubra* were relatively equal [65]. However, the tannins/non-tannins ratio was about two times lower for *Q. rubra* (presenting relatively more small phenolic molecules, possibly better gut-absorbable).

However, all those up-to-date analyses omit triterpenes. Specific compounds of this type were isolated from *Quercus* species since the 1960s (~210 works recorded in Chemical Abstracts). Thus far, no triterpenes or saponins were confirmed in acorns from *Q. petraea* or *Q. robur*, only in their wood [27,67,68,69]. Some unusual and bioactive triterpenes were isolated from acorns of oriental oaks [26,70,71,72]. Additionally, it was defined that *Q. ilex* acorn triterpenes were present only in hulls but not in flesh [73].

Compared with Mediterranean oak acorns, particularly those of *Q. ilex* and *Q. rotundifolia*, which are generally characterized by a higher lipid contribution and a high proportion of oleic acid, the Central European acorns investigated in the present study showed lower lipid contents in *Q. robur* and *Q. petraea*, whereas *Q. rubra* had a comparatively higher lipid level [39,47]. Nevertheless, all three Central European species exhibited a favourable fatty acid profile dominated by unsaturated fatty acids, with oleic acid accounting for 48.11–59.73% of total fatty acids, suggesting their potential relevance as an energy-providing dietary component for pigs [39,50,52]. Their main nutritional limitations were the relatively low protein contribution and the imbalance of essential amino acids, particularly the low concentrations of sulfur-containing amino acids, which may need to be considered when formulating balanced diets containing acorns [54,57,59]. At the same time, the nutritional and practical value of acorns depends not only on their proximate composition but also on the content and composition of tannins and other bioactive compounds, which may differ among *Quercus* species [11,12]. Therefore, on the basis of the present results and the literature, Central European oak acorns should not be regarded simply as a less fatty alternative to Mediterranean acorns, but as a distinct, locally available and species-dependent feed resource with potential relevance to pig nutrition. Therefore, from a practical perspective, acorns with a naturally low content of tannins and triterpenes may represent a more favourable starting material for further investigation of their potential relevance to indoor pig nutrition. Subsequent processing to improve nutritional value by reducing the content of tannins and triterpenes [28] would generate additional costs that would need to be economically justified.

## 4. Materials and Methods

### 4.1. Plant Material

Mature acorns of *Quercus petraea*, *Quercus robur* and *Quercus rubra* were collected on 5 October 2025 from healthy mature trees (approximately 100 years old) growing in Szczytnicki Park, Wrocław, Poland. Acorns were collected randomly from the ground beneath the tree canopy immediately after natural seed fall to ensure full physiological maturity. Only intact, undamaged and disease-free acorns were selected for further analyses. Acorns collected from multiple mature trees of each *Quercus* species were pooled to obtain one representative composite sample per species for subsequent analyses.

After collection, the acorns were pre-dried in a food air dehydrator at 35 °C for 48 h. The pre-dried material was subsequently ground using an A11 Basic laboratory mill (IKA, Staufen, Germany) to obtain a homogeneous powder for subsequent analyses.

### 4.2. Proximate Composition

The proximate composition of the pre-dried, fresh-like acorn material, including dry matter, crude ash, crude protein, crude fat and crude fibre, was determined according to the methods of the Association of Official Analytical Chemists [74]. Dry matter, crude ash, nitrogen, crude fat and crude fibre were determined according to AOAC methods 930.15, 942.05, 984.13, 920.39 and 978.10, respectively. Crude protein was calculated as nitrogen × 6.25. The proximate composition values reported by the laboratory were used as the as-is values for the pre-dried, fresh-like material. For nutritional comparison, these values were additionally recalculated on a dry-matter basis using the determined dry matter content. For each *Quercus* species, the representative composite sample obtained from acorns collected from multiple trees was used for proximate composition analysis.

### 4.3. Amino Acid Composition

The analyzed samples were acid-hydrolysed and placed in an AAA400 automatic amino acid analyser (INGOS s.r.o., Prague, Czech Republic). A two-wavelength photometer (440 and 570 nm) was employed as a detector. The length of the column packed with ion exchanger Ostion LG ANB (INGOS s.r.o., Prague, Czech Republic) was 350 × 3.7 mm, and the column temperature was maintained at 40–70 °C, and the detector temperature was kept at 121 °C. The amino acids were quantified using the ninhydrin method. Glutamine and asparagine were expressed as glutamic acid and aspartic acid, respectively. No analysis was carried out for tryptophan. Calculations were performed with the computer program Chromulan (Pikron s.r.o., Prague, Czech Republic). The amino acid composition was determined in duplicate using the representative composite sample obtained for each *Quercus* species [75].

### 4.4. Fatty Acid Composition

Lipids were extracted from pre-dried and ground fresh-like acorn material according to a modified Folch procedure. Briefly, 1.00 g of each sample was extracted with 20 mL of a chloroform–methanol mixture (2:1, *v*/*v*) for 24 h at 37 °C using a rotary shaker. The extracts were filtered through a Büchner funnel, and the solvent was removed under reduced pressure. The obtained lipid fraction was dissolved in methyl tert-butyl ether (MTBE), transferred to a pear-shaped 50 mL flask and evaporated to dryness.

Fatty acid methyl esters (FAMEs) were prepared by alkaline hydrolysis with 1 M KOH in methanol under reflux, followed by methylation using boron trifluoride etherate. After the addition of distilled water, FAMEs were extracted with ligroin, the organic phase was evaporated to dryness, and the residue was dissolved in 1.5 mL of cyclohexane.

Fatty acid analysis was performed in triplicate using the representative composite sample obtained for each *Quercus* species. Chromatographic analysis was carried out using an Agilent 7890A GC system equipped with a flame ionization detector (FID) (Agilent Technologies, Santa Clara, CA, USA) and a Zebron FAME capillary column (60 m × 0.25 mm i.d., 0.20 μm film thickness). Carrier gas H_2_, constant flow 2 mL/min, inlet temperature 280 °C. Oven program: 120 °C (hold 1 min), 1.2 °C/min to 220 °C (hold 0 min), 6 °C/min to 270 °C (hold 1 min). FID temperature 280 °C. Individual FAMEs were identified by comparison of retention times with Supelco 37 Component FAME Mix (CRM 47885) and, additionally, authentic standards of methyl laurate, myristate, palmitate, stearate and linoleate.

### 4.5. LCMS Examination

Homogenized whole-acorn pre-dried samples were extracted with 70% aqueous methanol in a sample-to-solvent ratio of 100 mg per 1.0 mL with the use of an ultrasonic bath (15 min, 100% power) [35,76]. Samples were vigorously shaken, centrifuged, filtered through a 0.22 µm PVDF membrane, and diluted in 70% aq. methanol and immediately subjected to UHPLC-qTOF-HRMS and -MS2 analysis to avoid redox-dependent tannin degradation. The instrumentation and procedures were as in the previous study [76] except that the calibration agent was sodium formate clusters.

Briefly, the UHPLC Ultimate 3000 apparatus (Thermo Fisher Scientific, Waltham, MA, USA) equipped with a Kinetex C18 column (150 × 2.1 mm, core–shell grain 2.6 μm; Phenomenex, Torrance, CA, USA) worked in a connection to an ESI-qTOF Compact mass spectrometer (Bruker Daltonics, Bremen, Germany). The following screening gradient was used: eluent A (water/formic acid, 99.9/0.1, V/V), eluent B (acetonitrile/formic acid, 99.9/0.1, V/V), program (B in A): 0–1 min (2% B), 1–31 min (2–100% B), 31–35.5 min (100% B), 35.5–37 min (100–2% B), 37–40 min (2% B). UHPLC parameters: flow 0.3 mL/min, injection volume 5 μL, column oven temperature 30 °C. ESI-MS parameters: calibration with the sodium formate (in dead volume of each analysis), negative mode, scan range 200–2200 *m*/*z*, dry gas (N_2_) 7.0 L/min, temperature 200 °C, capillary voltage 2.2 kV, ion energy 5 eV, collision energy automatic adjusted. Data were processed using Data Analysis 4.3 software (Bruker Daltonics, Bremen, Germany).

LCMS tentative identification was based on three stages. First, a consensus between low error and low mSigma (monoisotopic matching) parameters was established to propose a formula. Then, the formula was verified with the MS^2^ fragmentation (if available), and specific losses were analyzed. The results are collected in Table 4, alongside the third stage—Metabolomics Standards Initiative (MSI) [DOI 10.1007/s11306-007-0082-2] identification levels. Chemical analytical standards for a selected few common substances with level 1 were from Merck (Darmstadt, Germany). Putatively identified substances with level 2 (MS2 and/or UV data available, formula and fragmentation pathway consistent with already identified substance with MSI level 1, in any oak: papers and structures analyzed manually via Chemical Abstracts, supported by MS-Dial databases) and level 3 (MS2 and/or UV data not available, formula and chromatographic mobility consistent with already identified substance with MSI level 1, or previously in this study with better level 2) are treated as detailed fingerprint information, not definitive identification. It was assumed that if a specific species is selected for feeding, the most important components will be isolated and fully identified. Compounds—even with MS2 data, but without a solid identification ground—were classified as level 4 (unidentified).

### 4.6. Minerals and Heavy Metals Analysis

The concentrations of mineral elements and heavy metals were determined following microwave-assisted wet digestion. Briefly, 0.5 g of finely ground acorn sample was transferred into Teflon digestion vessels, followed by the addition of 2 mL of concentrated sulfuric acid (98%) and 6 mL of concentrated nitric acid (65%). The samples were mineralized using a Mars 6 microwave digestion system (CEM Corporation, Matthews, NC, USA) according to the manufacturer’s recommended program. After cooling, 2 mL of 30% hydrogen peroxide was added to each digest, and the samples were left to stand for 2 h. The digests were quantitatively transferred into 50 mL volumetric flasks, the digestion vessels were rinsed with distilled water, and the rinsing solutions were combined with the digests. Finally, the solutions were diluted to volume with distilled water.

The concentrations of the analyzed mineral elements and heavy metals were determined using a 4210 MP-AES microwave plasma–atomic emission spectrometer (Agilent Technologies, Santa Clara, CA, USA). The analyzed elements included P, Ca, K, Mg, Na, Fe, Zn, Cu, Mn, B, Mo, Cd, Pb, Ni, Cr, Sr and Al. The concentrations were expressed on a fresh-like basis, consistent with the presentation of the other chemical composition data.

Nitrates were determined separately according to the method of Nowosielski [77], with the extraction procedure adapted to acorn material. Briefly, 0.5 g of dried, ground acorn material was extracted with 100 mL of 2% acetic acid. The extract was shaken for 40 min, after which nitrate concentration was determined using a CPI-505 ion metre equipped with a nitrate ion-selective electrode. The electrode was a double-junction silver chloride electrode, with 1 M KCl as the inner filling solution and (NH_4_)_2_SO_4_ as the outer filling solution.

### 4.7. Statistical Analysis

Statistical analyses were performed using R software (R Core Team, 2025). Prior to statistical testing, the assumptions of normality and homogeneity of variance were assessed using the Shapiro–Wilk and Levene’s tests, respectively. Depending on the distributional properties of the data, one-way analysis of variance (ANOVA), generalized least squares (GLS) models with variance weighting, or generalized linear models (GLMs) with a Gamma distribution and logarithmic link were applied. For the amino acid data (Table 2), post hoc comparisons were performed using Duncan’s multiple range test. For the mineral and heavy metal data (Table 5), post hoc pairwise comparisons were performed using Tukey’s test based on estimated marginal means (EMMs), with Satterthwaite adjustment of degrees of freedom where required. Differences were considered statistically significant at *p* < 0.05. Statistical analyses were performed using the nlme, emmeans, and multcomp packages in R. Because each species was represented by one composite sample, the analytical replicates were not considered independent biological replicates. Therefore, the statistical results were interpreted with caution and are presented as exploratory/descriptive comparisons rather than evidence of biological variation among trees or populations.

## 5. Conclusions

The present study revealed differences in the chemical composition of acorns collected from three *Quercus* species. Each species exhibited distinct nutritional characteristics, with *Q. rubra* characterized by the highest crude fat and oleic acid contents, *Q. petraea* by the highest concentrations of amino acids and macroelements, and *Q. robur* by a comparatively balanced chemical profile.

The low concentrations of heavy metals support the potential use of appropriately selected acorns as a local feed material, provided that routine quality and safety control is applied.

Overall, the results indicate that acorns from the investigated Central European *Quercus* species represent a locally available feed resource with potential relevance to pig nutrition. Considering the overall chemical profile, including the nutritional composition and the relative occurrence of phytochemical compounds, *Q. robur* may represent a promising candidate for further investigation. However, this selection is hypothesis-generating and should be considered preliminary. Dedicated feeding, digestibility, bioavailability and safety studies using independently collected and processed samples are required before any species-specific feeding recommendation can be made.

## Figures and Tables

**Table 1:** Proximate composition of acorns (%).

Parameter	*Q. rubra*	*Q. robur*	*Q. petraea*
As-is basis			
Dry matter	67.49	65.37	57.16
Crude ash	1.28	1.26	1.24
Crude protein	3.27	3.24	3.20
Crude fat	9.04	2.62	2.10
Crude fibre	16.63	5.76	5.73
Dry-matter basis			
Crude ash	1.90	1.93	2.17
Crude protein	4.85	4.96	5.60
Crude fat	13.39	4.01	3.67
Crude fibre	24.64	8.81	10.02

Data are expressed on an as-is basis for the pre-dried, fresh-like acorn material. Dry-matter values were calculated from the determined dry matter content.

**Table 2:** The crude protein and amino acid composition of acorns.

	*Q. rubra*	*Q. petraea*	*Q. robur*	
Amino Acid (mg/g Fresh-Like Matter.)				*p*-Value (ANOVA)
ASP	3.440 ± 0.000 ^c^	4.660 ± 0.000 ^a^	3.850 ± 0.028 ^b^	0.0000
THR	1.305 ± 0.007 ^b^	1.525 ± 0.007 ^a^	1.295 ± 0.007 ^c^	0.0001
SER	1.520 ± 0.000 ^b^	1.790 ± 0.000 ^a^	1.515 ± 0.007 ^c^	0.0000
GLU	5.555 ± 0.049 ^b^	6.120 ± 0.014 ^a^	5.385 ± 0.092 ^c^	0.0025
PRO	1.575 ± 0.035 ^c^	2.265 ± 0.092 ^a^	1.765 ± 0.106 ^b^	0.0079
GLY	1.545 ± 0.007 ^c^	1.885 ± 0.007 ^a^	1.600 ± 0.014 ^b^	0.0001
ALA	1.670 ± 0.014 ^c^	1.965 ± 0.007 ^a^	1.675 ± 0.021 ^b^	0.0005
CYS	0.165 ± 0.049 ^a^	0.085 ± 0.035 ^a^	0.120 ± 0.057 ^a^	0.3723
VAL	1.715 ± 0.007 ^c^	2.080 ± 0.057 ^a^	1.830 ± 0.014 ^b^	0.0038
MET	0.080 ± 0.000 ^c^	0.285 ± 0.007 ^a^	0.210 ± 0.028 ^b^	0.0027
ILE	1.380 ± 0.014 ^c^	1.660 ± 0.014 ^a^	1.440 ± 0.014 ^b^	0.0006
LEU	2.605 ± 0.007 ^c^	3.080 ± 0.014 ^a^	2.705 ± 0.007 ^b^	0.0000
TYR	0.790 ± 0.000 ^c^	0.990 ± 0.000 ^a^	0.910 ± 0.014 ^b^	0.0003
PHE	1.300 ± 0.000 ^c^	1.785 ± 0.007 ^a^	1.590 ± 0.014 ^b^	0.0000
HIS	0.865 ± 0.064 ^a^	1.155 ± 0.191 ^a^	1.095 ± 0.035 ^a^	0.1711
LYS	1.965 ± 0.007 ^b^	2.330 ± 0.085 ^a^	1.855 ± 0.035 ^c^	0.0061
ARG	2.295 ± 0.007 ^c^	2.870 ± 0.057 ^a^	2.655 ± 0.049 ^b^	0.0021
Sum of amino acids	29.77 ± 0.060 ^c^	36.53 ± 0.280 ^a^	31.50 ± 0.560 ^b^	0.0000

Values are expressed as mean ± SD of analytical replicates (*n* = 2). Different superscript letters within a row indicate statistically significant differences between the analyzed composite samples according to Duncan’s multiple range test (*p* < 0.05). As each *Quercus* species was represented by one composite sample, these comparisons are exploratory and do not represent independent biological replication.

**Table 3:** Fatty acid composition of acorns.

Fatty Acid	Abbreviation	Retention Time (min)	*Q. petraea*	*Q. rubra*	*Q. robur*	Class
Lauric acid	12:0	7.24	0.04 ± 0.01	0.04 ± 0.00	0.02 ± 0.00	SFA
Myristic acid	14:0	12.06	0.17 ± 0.01	0.11 ± 0.01	0.09 ± 0.00	SFA
Pentadecanoic acid	15:0	15.39	0.08 ± 0.01	0.02 ± 0.00	0.06 ± 0.01	SFA
Palmitic acid	16:0	19.28	12.38 ± 0.03	9.01 ± 0.05	13.52 ± 0.11	SFA
Palmitoleic acid	16:1Δ9c	20.91	0.15 ± 0.01	0.17 ± 0.01	0.25 ± 0.01	MUFA
Heptadecanoic acid	17:0	23.6	0.10 ± 0.01	0.09 ± 0.01	0.09 ± 0.01	SFA
Stearic acid	18:0	28.23	2.06 ± 0.02	1.32 ± 0.19	1.83 ± 0.03	SFA
Oleic acid	18:1Δ9c	29.49	48.56 ± 0.27	59.73 ± 0.10	48.11 ± 0.13	MUFA
Linolelaidic acid	18:2Δ9,12t	31.36	0.38 ± 0.04	0.42 ± 0.02	0.38 ± 0.03	PUFA
Linoleic acid	18:2Δ9,12c	32.44	31.14 ± 0.22	27.46 ± 0.21	32.08 ± 0.27	PUFA
α-Linolenic acid	18:3Δ9,12,15c	36.2	3.30 ± 0.07	0.38 ± 0.01	1.85 ± 0.02	PUFA
Arachidic acid	20:0	37.91	0.46 ± 0.00	0.42 ± 0.01	0.48 ± 0.01	SFA
Gondoic acid	20:1 Δ11c	39	0.39 ± 0.01	0.49 ± 0.00	0.46 ± 0.01	MUFA
Heneicosanoic acid	21:0	42.78	0.06 ± 0.01	0.03 ± 0.01	0.07 ± 0.01	SFA
Behenic acid	22:0	47.62	0.37 ± 0.01	0.17 ± 0.01	0.40 ± 0.01	SFA
Tricosanoic acid	23:0	52.36	0.09 ± 0.01	0.03 ± 0.00	0.09 ± 0.00	SFA
Lignoceric acid	24:0	57.04	0.28 ± 0.01	0.10 ± 0.01	0.23 ± 0.02	SFA
Total fatty-acid classes						
SFA			16.09 ± 0.05	11.35 ± 0.16	16.87 ± 0.17	SFA
MUFA			49.10 ± 0.27	60.40 ± 0.11	48.83 ± 0.14	MUFA
PUFA			34.82 ± 0.31	28.25 ± 0.22	34.30 ± 0.29	PUFA

Values are expressed as mean ± SD of analytical replicates (*n* = 3). The reported variability represents analytical repeatability of the representative composite sample for each *Quercus* species and does not represent independent biological replications.

**Table 4:** LCMS fingerprints of *Quercus petraea*, *Quercus robur* and *Quercus rubra* acorn 70% aqueous methanol extracts (QPet_70M, QRob_70M, QRub_70M). Negative mode.

RT [min]	Compound Name ([M−H]^−^ *m*/*z*, RT)	UV_max_ [nm]	Neutral Formula [M]	|err| [ppm]	mSigma	MS2	Compound Identification/ Tentative Identification	MSI Level	Class	QPet_70M [RA%]	QRob_70M [RA%]	QRub_70M [RA%]
1.06	179, 1.06 min	―	C_6_H_12_O_6_	1.2	6.6	overl.	glucose	1	SUG	100.0	90.6	10.4
1.08	341/387, 1.08 min	―	C_12_H_22_O_11_	2.6	3.0	overl.	sucrose	1	SUG	96.9	57.9	42.5
1.14	191, 1.14 min	―	C_6_H_8_O_7_	2.4	2.0	overl.	citric/isocitric acid	2	SAC	84.6	100.0	44.5
1.39	191, 1.39 min	―	C_6_H_8_O_7_	0.3	2.7	overl.	citric/isocitric acid	2	SAC	87.2	85.2	41.7
1.50	169.01, 1.50 min	272	C_7_H_6_O_5_	1.8	7.1	125	gallic acid isomer	2	PAC	―	1.5	―
1.65	169.01, 1.65 min	272	C_7_H_6_O_5_	0.1	4.7	125	gallic acid [GA]	1	PAC	5.2	9.8	88.7
3.21	153, 3.21 min	―	C_7_H_6_O_4_	0.5	2.0	152, 124	protocatechuic acid	1	PAC	―	―	7.4
5.38	169.05, 5.38 min	―	C_8_H_10_O_4_	0.1	0.7	137 [M−MeOH−H]^−^	*p*-methyl gallic alcohol, CAS 63542-40-5 or isomer	3	PAL	―	―	37.6
8.00	337, 8.00 min	―	C_14_H_26_O_9_	1.7	6.4	175 [M−Hex−H]^−^ 157 [M−Hex−H_2_O−H]^−^ 129 [M−Hex−H_2_O−CO−H]^−^	unidentified hexoside	4	SUG	―	―	2.1
8.30	183, 8.30 min	178	C_8_H_8_O_5_	0.7	0.9	124	methyl gallate	2	PAC	2.2	38.0	22.6
8.39	483, 8.39 min	178	C_20_H_20_O_14_	0.2	27.5	overl.	digalloylglucose isomer	3	TAN	―	0.8	―
8.78	483, 8.78 min	178	C_20_H_20_O_14_	2.8	27.1	313, 271, 211, 169	digalloylglucose isomer	2	TAN	―	1.4	2.3
8.94	783 (z = −2)/1567, 8.94 min	257, 276	C_68_H_48_O_44_	2.0	58.0	785, 765, 613, 597, 301, 273, 169	hydrolyzable tannin, e.g., oenothein B/sanguiin H	2 *	TAN	―	―	13.4
8.94	337, 8.94 min	―	C_14_H_26_O_9_	1.7	59.8	overl.	unidentified hexoside	4	SUG	―	―	0.5
9.07	387, 9.07 min	―	C_18_H_28_O_9_	0.9	4.8	163 [M−Hex−H_2_O−CO_2_−H]^−^	tuberonic acid hexoside or isomer	2	JAC	7.0	2.9	0.7
9.07	467, 9.07 min	―	C_20_H_20_O_13_	0.2	45	―	neocretanin	2	TAN	―	―	0.7
9.24	321, 9.24 min	257, 278	C_14_H_10_O_9_	2.9	7.0	overl.	galloyl gallate	3	TAN	―	―	1.2
9.24	635, 9.24 min	―	C_27_H_24_O_18_	0.0	12.5	overl.	trigalloylglucose isomer	3	TAN	―	―	0.8
9.27	387, 9.27 min	―	C_18_H_28_O_9_	1.4	6.4	163 [M−Hex−H_2_O−CO_2_−H]−	tuberonic acid hexoside or isomer	2	JAC	5.1	2.4	1.4
9.39	305, 9.39 min	―	C_12_H_18_O_7_S	1.0	0.8	225 [M−SO_3_−H]^−^, 165	tuberonic acid sulfate	2 *	JAC	13.0	6.5	0.2
9.53	387, 9.53 min	―	C_18_H_28_O_9_	1.1	18.6	247, 207, 163	tuberonic acid hexoside or isomer	2	JAC	1.8	0.6	―
9.60	387, 9.60 min	―	C_18_H_28_O_9_	1.0	2.1	289, 247, 207, 163	tuberonic acid hexoside or isomer	2 *	JAC	14.7	3.7	22.3
9.67	307, 9.67 min	―	C_12_H_20_O_7_S	1.1	5.4	227 [M−SO_3_−H]^−^, 167	dihydrotuberonic acid sulfate	2	JAC	3.0	1.2	―
9.70	859 (z = −2)/1719, 9.70 min	278	C_75_H_52_O_48_	3.4	34.4	overl.	hydrolyzable tannin	3	TAN	―	―	1.4
9.75	631, 9.75 min	―	C_26_H_32_O_18_	1.1	10.2	overl.	unidentified	4	UNI	―	―	0.9
9.82	785, 9.82 min	278	C_34_H_26_O_22_	0.5	3.1	615, 463, 301, 275, 249	(HHDP + digalloyl) glucose	2	TAN	―	―	3.1
9.88	389, 9.88 min	―	C_18_H_30_O_9_	0.6	6.9	329 [M−AA−H]^−^	unidentified	4	UNI	3.9	1.5	0.7
9.95	169.01, 9.95 min	278	C_7_H_6_O_5_	1.0	2.3	―	trihydroxybenzoic acid, GA isomer	3	PAC	―	―	8.9
9.95	476 (z = −2)/953, 9.95 min	―	C_41_H_30_O_27_	3.8	71.7	overl.	hydrolyzable tannin, e.g., HHDP + trigalloyl glucose	3	TAN	―	―	0.7
10.07	387, 10.07 min	―	C_18_H_28_O_9_	0.9	6.4	207 [M−Hex−H]^−^ 163 [M−Hex−CO_2_−H]^−^	tuberonic acid hexoside or isomer	2 *	JAC	5.7	1.7	0.8
10.07	635, 10.07 min	―	C_27_H_24_O_18_	0.0	2.0	465, 313, 295, 169	trigalloylglucose isomer	2	TAN	―	―	4.2
10.11	463, 10.11 min	254	C_20_H_16_O_13_	2.0	7.8	301 [M−Hex−H]^−^	ellagic acid glucoside	2	TAN	9.9	11.4	0.8
10.23	225, 10.23 min	―	C_12_H_18_O_4_	0.6	2.1	147	tuberonic/hydroxyjasmonic acid	2	JAC	4.4	1.8	―
10.28	631, 10.28 min	278	C_26_H_32_O_18_	0.7	4.2	429, 169	unidentified tannin	3	TAN	―	―	5.0
10.33	635, 10.33 min	―	C_27_H_24_O_18_	1.7	11.3	overl.	trigalloylglucose isomer	2	TAN	―	1.5	―
10.35	225, 10.35 min	―	C_12_H_18_O_4_	1.4	2.3	147	tuberonic/hydroxyjasmonic acid	2 *	JAC	21.9	9.0	1.7
10.42	469, 10.42 min	275	C_20_H_22_O_13_	2.0	0.6	169	cretanin, CAS 64121-96-6 or isomer	2 *	TAN	―	―	100.0
10.48	635, 10.48 min	―	C_27_H_24_O_18_	2.0	8.6	―	trigalloylglucose isomer	3	TAN	―	1.0	―
10.61	541 (z = −2)/1083/933, 10.61 min	280	C_48_H_28_O_30_	2.3	7.3	―	hydrolyzable tannin, e.g., punicalagin or isomer	3	TAN	2.7	0.4	―
10.69	227, 10.69 min	―	C_12_H_20_O_4_	1.2	10.2	―	unidentified	4	UNI	2.0	0.6	―
10.72	607, 10.72 min	―	C_27_H_28_O_16_	0.8	11.4	481, 275, 169	unidentified tannin, (not a flavonoid dihexoside)	3	TAN	―	―	1.4
10.97	433, 10.97 min	254	C_19_H_14_O_12_	0.9	1.2	301 (ellagic acid)	ellagic acid pentoside	3	TAN	18.6	16.5	0.5
11.20	541 (z = −2)/1083/933, 11.20 min	―	C_48_H_28_O_30_	2.7	12.2	―	hydrolyzable tannin, e.g., punicalagin or isomer	3	TAN	2.6	0.5	―
11.21	335, 11.21 min	―	C_15_H_12_O_9_	2.6	2.0		methyl(galloyl gallate) isomer	3	TAN	―	5.3	1.5
11.23	447, 11.23 min	―	C_20_H_16_O_12_	0.3	3.6	300 [M−dHex−H]^−^	ellagic acid deoxyhexoside	2	TAN	3.0	1.0	―
11.37	787, 11.37 min	276	C_34_H_28_O_22_	1.3	4.9	617, 465, 447, 169	tetragalloyl glucose		TAN	―	1.7	8.6
11.56	301, 11.56 min	254	C_14_H_6_O_8_	1.9	4.7	229	ellagic acid	1	TAN	44.9	36.0	6.8
11.67	621, 11.67 min	276	C_27_H_26_O_17_	0.5	5.9	469, 313, 169	115397-24-5 or isomer	2	TAN	―	―	7.2
11.89	476 (z = −2)/953, 11.89 min	278	C_41_H_30_O_27_	0.2	4.8	169	hydrolyzable tannin, e.g., HHDP + trigalloyl glucose	2	TAN	―	7.0	2.2
11.90	621, 11.90 min	278	C_27_H_26_O_17_	0.5	8.9	469, 169	115397-24-5 or isomer	2	TAN	―	―	7.9
11.97	860 (z = −2)/1721, 11.97 min	280	C_75_H_54_O_48_	1.6	13.7	633, 465, 301, 169	hydrolyzable tannin, e.g., rugosin E-type	2 *	TAN	―	―	25.7
12.09	469 (z = −2)/939, 12.09 min	―	C_41_H_32_O_26_	1.3	6.9	169	pentagalloyl glucose	2	TAN	―	9.8	7.3
12.12	447, 12.12 min	―	C_20_H_16_O_12_	0.5	27.2	―	ellagic acid deoxyhexoside	2	TAN	―	0.8	―
12.20	335, 12.20 min	280	C_15_H_12_O_9_	1.2	3.2	183	methyl(galloyl gallate) isomer	2	TAN	―	11.4	3.4
12.32	476 (z = −2)/953, 12.29 min	―	C_41_H_30_O_27_	0.4	18.5	―	hydrolyzable tannin, e.g., HHDP + trigalloyl glucose	3	TAN	―	0.7	0.4
13.06	321, 13.06 min	―	unidentified	-	-	152	unidentified	4	UNI	5.6	1.1	0.2
13.31	665/711, 13.31 min	―	C_36_H_58_O_11_	1.2	4.7	503 [M−Hex−H]^−^	sericoside, arjunglucoside I, quadranoside III or isomer	2 *	TTP	11.7	―	―
13.61	517, 13.61 min	―	C_30_H_46_O_7_	0.3	43.9	―	bartogenic acid isomer	3	TTP	0.3	―	―
13.68	517, 13.68 min	―	C_30_H_46_O_7_	0.0	41.5	―	bartogenic acid isomer	3	TTP	0.8	―	―
13.81	517, 13.81 min	―	C_30_H_46_O_7_	0.2	20.7	―	bartogenic acid isomer	3	TTP	0.6	―	―
14.19	517, 14.19 min	―	C_30_H_46_O_7_	1.6	8.9	403, 353	bartogenic acid isomer	3	TTP	3.9	―	―
14.34	665/711, 14.34 min	―	C_36_H_58_O_11_	1.3	1.8	503 [M−Hex−H]^−^	sericoside, arjunglucoside I, quadranoside III or isomer	2 *	TTP	8.2	―	―
14.38	517, 14.38 min	―	C_30_H_46_O_7_	0.1	26.9	―	bartogenic acid isomer	3	TTP	0.9	―	―
14.46	665/711, 14.46 min	―	C_36_H_58_O_11_	1.2	4.4	503 [M−Hex−H]^−^	sericoside, arjunglucoside I, quadranoside III or isomer	2	TTP	6.8	―	―
14.56	517, 14.56 min	―	C_30_H_46_O_7_	0.0	11.5	407, 403 < 353	bartogenic acid isomer	2	TTP	1.9	―	―
14.59	709, 14.59 min	―	C_36_H_56_O_11_	0.6	49.4	501 [M−Hex−H]^−^	triterpene hexoside	3	TTP	3.5	―	―
14.64	563/517, 14.64 min	―	C_30_H_46_O_7_	1.7	8.6	425 > 407, 353	bartogenic acid isomer	2	TTP	2.9	―	―
14.75	709, 14.75 min	―	C_36_H_56_O_11_	1.2	16.9	overl.	triterpene hexoside	3	TTP	1.8	―	―
14.75	563/517, 14.75 min	―	C_30_H_46_O_7_	0.3	10.5	487 > 425	bartogenic acid isomer	2	TTP	5.5	―	―
14.87	563/517, 14.87 min	―	C_30_H_46_O_7_	0.2	14.5	487 < 425	bartogenic acid isomer	2	TTP	6.4	―	―
15.03	563/517, 15.03 min	―	C_30_H_46_O_7_	0.9	14.3	―	bartogenic acid isomer	3	TTP	0.7	―	―
15.28	665/711, 15.28 min	―	C_36_H_58_O_11_	1.3	14.5	―	sericoside, arjunglucoside I, quadranoside III or isomer	3	TTP	0.4	―	―
15.56	517, 15.56 min	―	C_30_H_46_O_7_	0.8	64.6	―	bartogenic acid isomer	3	TTP	0.7	―	―
15.75	517, 15.75 min	―	C_30_H_46_O_7_	1.8	32.4	―	bartogenic acid isomer	3	TTP	0.5	―	―
15.80	273, 15.80 min	―	C_15_H_14_O_5_	0.4	8.1	229, 189, 167, 151	phloretin or isomer	2	FLV	2.7	―	0.1
15.92	565/519, 15.92 min	―	C_30_H_48_O_7_	0.4	2.8	501	triterpene	2 *	TTP	5.7	―	―
15.99	665/711, 15.99 min	―	C_36_H_58_O_11_	0.6	46.2	―	sericoside, arjunglucoside I, quadranoside III or isomer	3	TTP	0.4	―	―
16.04	565/519, 16.04 min	―	C_30_H_48_O_7_	0.1	5.5	501, 489, 457, 407	triterpene	2 *	TTP	8.5	―	―
16.10	665/711, 16.10 min	―	C_36_H_58_O_11_	2.2	13.4	―	sericoside, arjunglucoside I, quadranoside III or isomer	3	TTP	0.6	―	―
16.18	563/517, 16.18 min	―	C_30_H_46_O_7_	1.7	143.9	―	bartogenic acid isomer	3	TTP	1.1	―	―
16.23	563/517, 16.23 min	―	C_30_H_46_O_7_	1.2	20.0	487, 469, 451, 407	bartogenic acid isomer	2	TTP	3.1	―	―
16.41	329, 16.41 min	―	C_18_H_34_O_5_	1.3	2.8	229, 211	trihydroxyoctadecenoic acid isomer	2	FAC	3.5	1.3	―
16.42	665/711, 16.42 min	―	C_36_H_58_O_11_	2.0	13.4	503 [M−Hex−H]^−^	sericoside, arjunglucoside I, quadranoside III or isomer	2	TTP	1.5	―	―
16.51	329, 16.51 min	―	C_18_H_34_O_5_	4.3	17.9	―	trihydroxyoctadecenoic acid isomer	3	FAC	0.4	―	―
16.55	665/711, 16.55 min	―	C_36_H_58_O_11_	0.2	7.2	503 [M−Hex−H]^−^	sericoside, arjunglucoside I, quadranoside III or isomer	2	TTP	3.3	―	―
16.74	563/517, 16.74 min	―	C_30_H_46_O_7_	0.6	29.8	―	bartogenic acid isomer	3	TTP	0.8	―	―
16.85	709, 16.85 min	―	C_36_H_56_O_11_	2.9	78.7	overl.	triterpene hexoside	3	TTP	0.7	―	―
16.85	563/517, 16.85 min	―	C_30_H_46_O_7_	0.1	11.2	451, 407	bartogenic acid isomer	2	TTP	3.1	―	―
17.15	503/549, 17.15 min	―	C_30_H_48_O_6_	2.8	2.2	no fragments despite the intensity	arjungenin or isomer	3 *	TTP	61.5	―	―
17.25	503/549, 17.25 min	―	C_30_H_48_O_6_	2.6	0.9	441	arjungenin or isomer	2 *	TTP	70.4	―	―
17.59	655, 17.59 min	―	C_37_H_52_O_10_	0.5	23.9	―	triterpene gallate, e.g., 2328024-63-9 or isomer	3	TTP	1.9	―	―
18.24	501/547, 18.24 min	―	C_30_H_46_O_6_	2.0	367.4	―	triterpene	3	TTP	23.6	―	―
18.25	517, 18.25 min	―	C_30_H_46_O_7_	2.3	3.4	447	bartogenic acid or its isomer	2	TTP	1.8	3.1	―
18.31	503/549, 18.31 min	―	C_30_H_48_O_6_	1.6	1.8	485, 409	arjungenin or its isomer	2	TTP	24.8	―	―
18.39	517, 18.39 min	―	C_30_H_46_O_7_	1.3	9.3	447	bartogenic acid or its isomer	2	TTP	6.1	6.8	―
18.41	503/549, 18.41 min	―	C_30_H_48_O_6_	2.4	4.3	485, 441	arjungenin or its isomer	2 *	TTP	63.4	―	―
18.48	655, 18.48 min	―	C_37_H_52_O_10_	0.9	3.0	503 [M−GA−H]−, 441	triterpene gallate, e.g., 2328024-63-9 or isomer	2	TTP	12.7	―	―
19.06	501/547, 19.06 min	―	C_30_H_46_O_6_	1.3	2.7	409	triterpene	3 *	TTP	21.3	―	―
19.16	501/547, 19.16 min	―	C_30_H_46_O_6_	1.9	1.5	421	triterpene	3 *	TTP	30.4	―	―
19.59	499, 19.59 min	―	C_30_H_44_O_6_	2.0	319.1	―	triterpene	3	TTP	0.6	―	―
19.78	487, 19.78 min	―	C_30_H_48_O_5_	0.1	8.2	―	triterpene	3	TTP	1.6	―	―
19.94	487, 19.94 min	―	C_30_H_48_O_5_	2.4	1.8	no fragments despite the intensity	triterpene	3	TTP	4.0	―	―
20.71	487, 20.71 min	―	C_30_H_48_O_5_	0.1	9.2	―	triterpene	3	TTP	1.0	―	―
20.82	487, 20.82 min	―	C_30_H_48_O_5_	0.0	2.3	469, 467, 457, 441, 439, 425, 423	triterpene	2	TTP	5.5	―	―
20.91	499, 20.91 min	―	C_30_H_44_O_6_	1.9	10.8	469, 453, 437, 409, 379	triterpene	2	TTP	8.0	―	―
21.55	487, 21.55 min	―	C_30_H_48_O_5_	1.7	32.8	―	triterpene	3	TTP	0.8	―	―
21.72	487, 21.72 min	―	C_30_H_48_O_5_	2.5	32.2	―	triterpene	3	TTP	0.6	―	―
21.89	487, 21.89 min	―	C_30_H_48_O_5_	0.7	7.2	―	triterpene	3	TTP	1.1	―	―

**Abbreviations:** AA—acetic acid loss, dHex—deoxyhexose loss, GA―gallic acid, Hex—hexose loss, overl.―overlied, RA—the relative area of peak, when the area of the largest one is calculated as 100%, RT―retention time, UV_max_―wavelength of ultraviolet light where an analyte shows its strongest absorbance. Compounds classes: FAC―fatty acid, FLV―flavonoid, JAC―jasmonic acid, PAC―phenolic acid, PAL―phenolic alcohol, SAC―simple organic acid, SUG―sugar/sugar derivative, TAN―tannin, TTP―triterpenoid, UNI―unidentified. Metabolomics Standards Initiative (MSI)-guided [DOI 10.1007/s11306-007-0082-2] identification levels: 1—confirmed with analytical standard, 2—putatively annotated compounds (without chemical reference standards, based upon physicochemical properties and/or spectral similarity with spectral libraries: first—Chemical Abstracts and second—MS-Dial databases; MS2 and/or UV spectrum data available), 3—putatively characterized compound classes (based upon a formula corresponding to already better identified substance; no MS2 data available), 4—unknown compounds. Distinctive compounds with MSI confidence level marked with and an asterisk have MS2 data presented in Supplementary Figure S3.

**Table 5:** Macroelement, microelement, heavy metal and nitrate contents of acorns from three *Quercus* species (mg/kg fresh-like matter).

Species	P	Ca	K	Mg	Na	Zn	Fe	Cu	B	Mn	Cd	Sr	Pb	Mo	Al	NO_3_
*Q. rubra*	98.0 ^a^	133.9 ^a^	539.5 ^c^	27.1 ^c^	21.4 ^a^	0.9 ^a^	3.6 ^a^	1.2 ^a^	6.7 ^a^	12.4 ^a^	0.3 ^a^	0.3 ^a^	2.3 ^a^	1.3 ^a^	1.1 ^b^	127
*Q. robur*	92.3 ^a^	83.2 ^c^	618.6 ^b^	36.1 ^b^	18.2 ^b^	1.1 ^a^	3.4 ^a^	1.2 ^a^	3.8 ^b^	6.7 ^c^	0.3 ^a b^	0.1 ^c^	2.3 ^a^	1.5 ^a^	0.9 ^b^	85
*Q. petraea*	106.0 ^a^	89.0 ^b^	700.5 ^a^	44.6 ^a^	16.2 ^c^	1.3 ^a^	3.8 ^a^	1.2 ^a^	3.3 ^b^	8.3 ^b^	0.2 ^b^	0.1 ^b^	2.3 ^a^	1.6 ^a^	1.5 ^a^	137

Values are expressed as mean ± SD of analytical replicates. Different superscript letters indicate statistically significant differences between the analyzed composite samples according to Tukey’s test (*p* < 0.05). As each *Quercus* species was represented by one composite sample, these comparisons are exploratory and do not represent independent biological replication.

## Data Availability

Research data is available from authors.

## Supplementary Materials

The following supporting information can be downloaded at: https://www.mdpi.com/article/10.3390/molecules31172967/s1, Figure S1: LCMS fingerprints of *Quercus petraea*, *Quercus robur* and *Quercus rubra* acorn 70% aqueous methanol extracts. Figure S2: PCA of normalized LCMS data for *Quercus petraea*, *Quercus robur* and *Quercus rubra* acorn 70% aqueous methanol extracts. Figure S3: A-O: MS2 spectra of distinctive constituents of analyzed *Quercus petraea*, *Quercus robur* and *Quercus rubra* acorn 70% aqueous methanol extracts.
