# Supplementary material for: Comprehensive Characterization of Acorns from Selected Quercus Species: Nutritional Composition, Phytochemical Profile and Potential Relevance to Swine Nutrition
Source: Molecules. 2026 Aug 25;31(17):2967. doi: 10.3390/molecules31172967 (PMC13567339; doi:10.3390/molecules31172967)
Supplement: Supplementary file 1 [file molecules-31-02967-s001.zip › molecules-4463069-supplementary.pdf]

Supplementary Materials to:

Comprehensive Characterization of Acorns from Selected *Quercus* Species: Nutritional Composition, Phytochemical Profile and Potential Relevance to Swine Nutrition

by:

Anna Zwyrzykowska-Wodzińska, Przemysław Bąbelewski, Anna M. Salejda, Bogdan Jarosz, Maciej Włodarczyk, Aleksandra Szmaja, Joanna Miedzianka, Marta Czaplicka, Patryk Jagoda, Zdenek Zadák, Anna Jankowska-Mąkosa and Damian Knecht

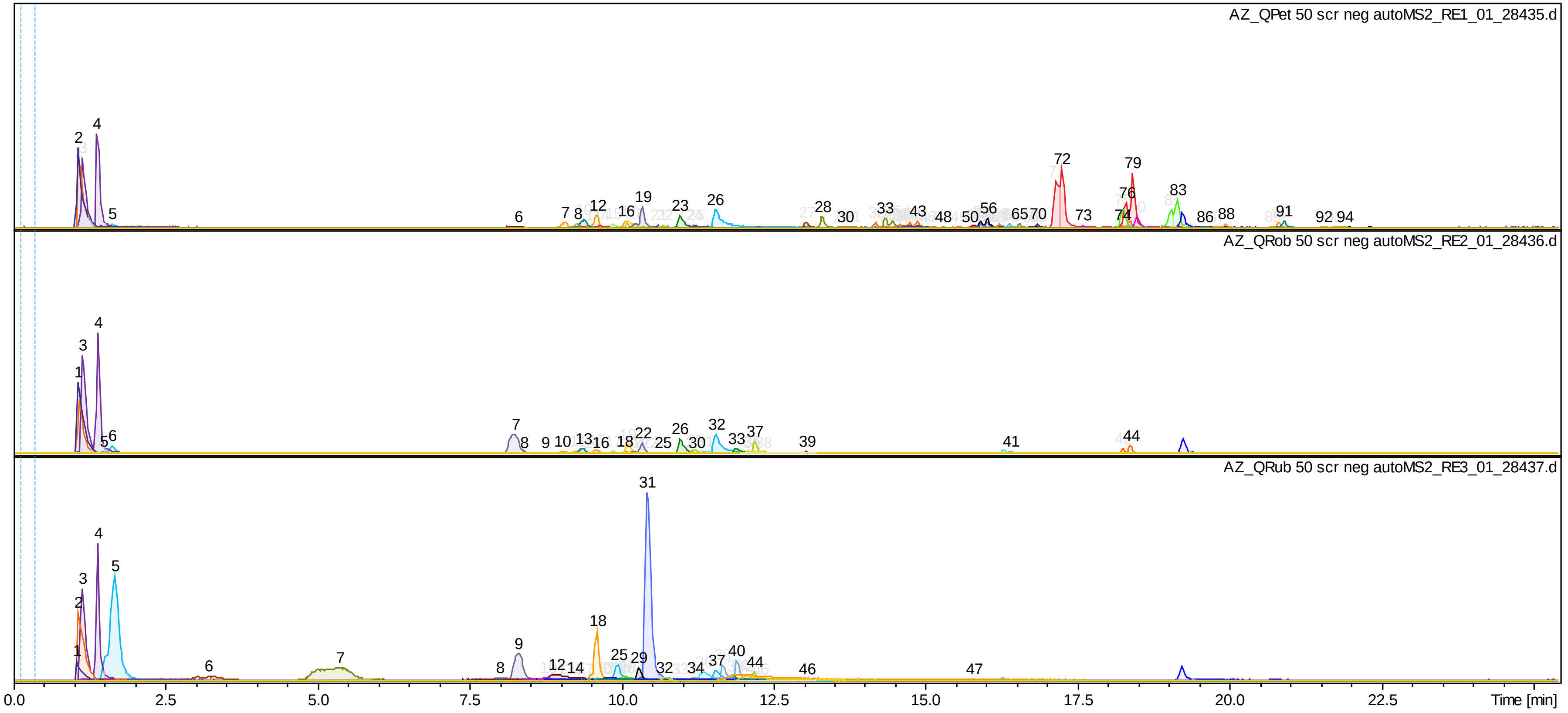

Figure S1: LCMS fingerprints of *Quercus petraea*, *Quercus robur* and *Quercus rubra* acorn 70% aqueous methanol extracts (QPet_70M, QRob_70M, QRub_70M) in order from top to bottom. Negative mode. The observed secondary metabolites were found in relatively the smallest amounts and concentrations in European/English oak acorns.

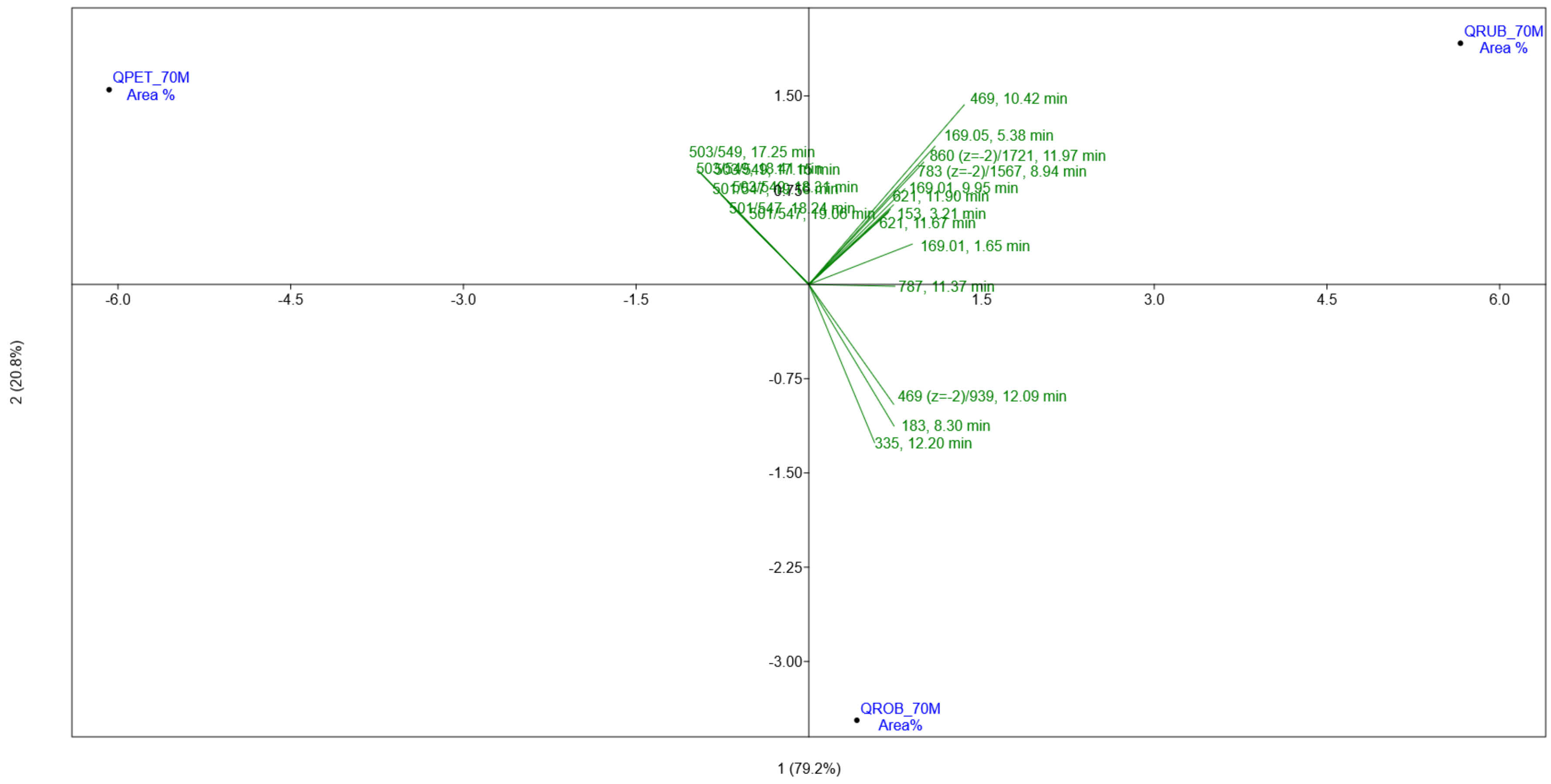

Figure S2: PCA of normalized LCMS data for *Quercus petraea*, *Quercus robur* and *Quercus rubra* acorn 70% aqueous methanol extracts (QPet_70M, QRob_70M, QRub_70M). Only the 20 most influential metabolites are shown for readability.

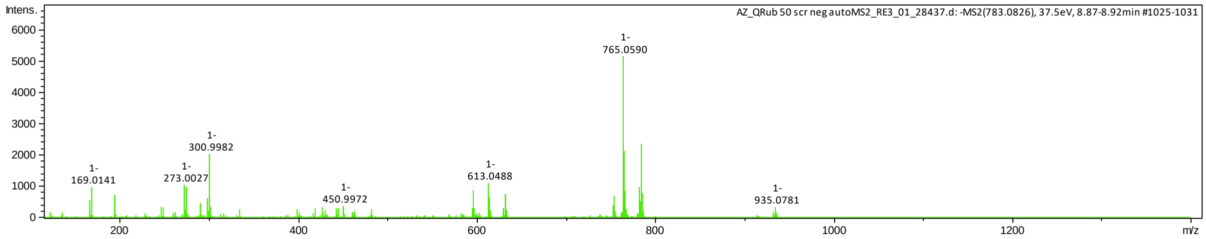

A 783 (z=-2)/1567, 8.94 min hydrolyzable tannin, e.g., oenothein B/sanguiin H

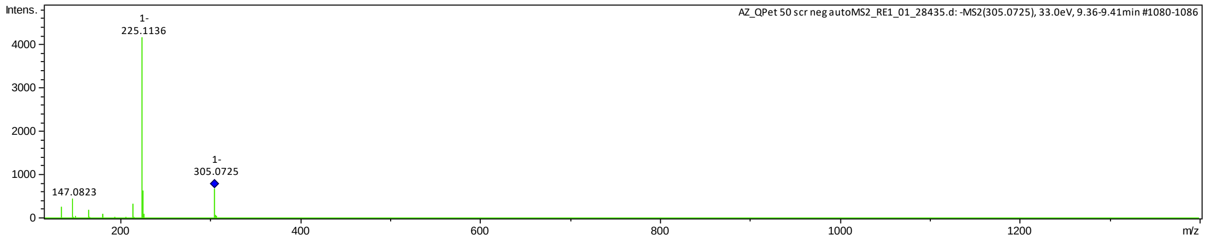

B 305, 9.39 min tuberonic acid sulfate

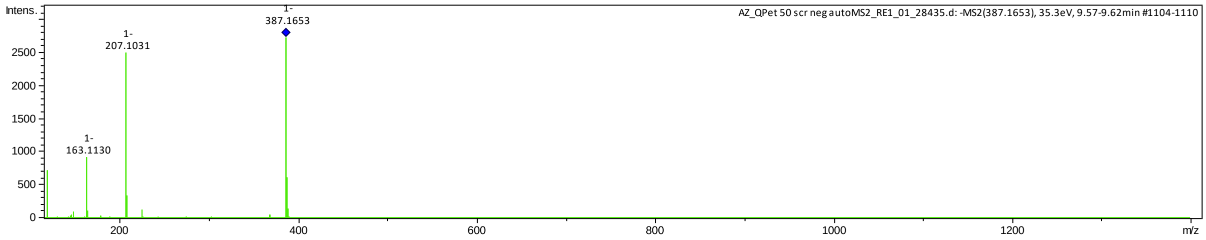

C 387, 9.60 min tuberonic acid hexoside or isomer

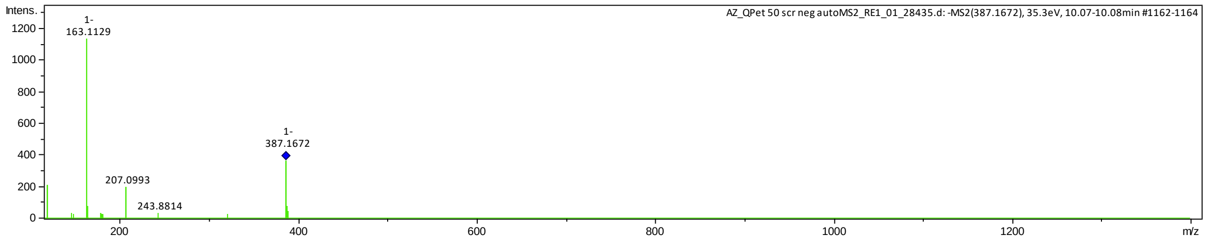

D 387, 10.07 min tuberonic acid hexoside or isomer

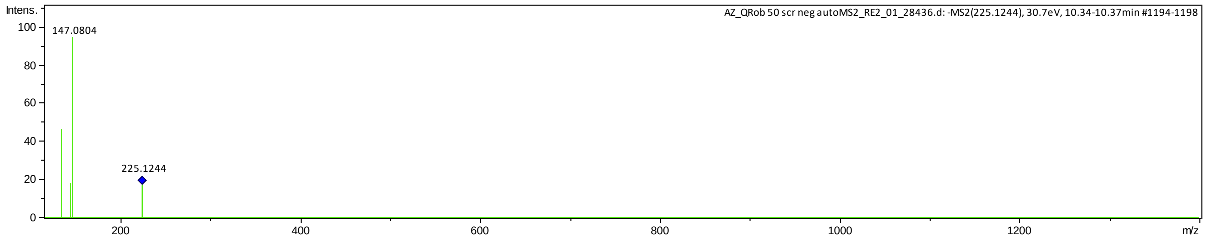

E 225, 10.35 min tuberonic/hydroxyjasmonic acid

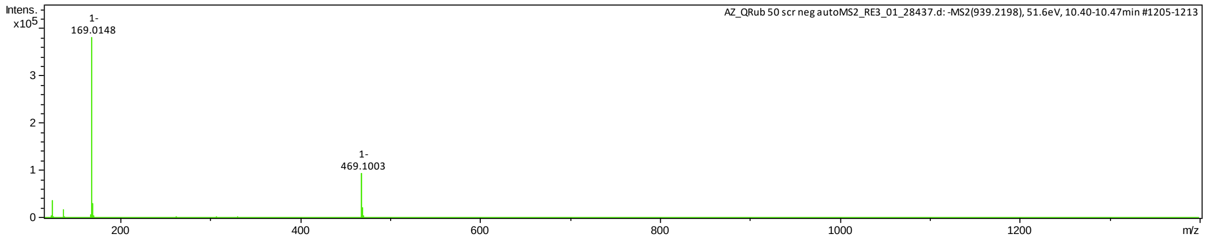

F 469, 10.42 min cretanin, CAS 64121-96-6 or isomer

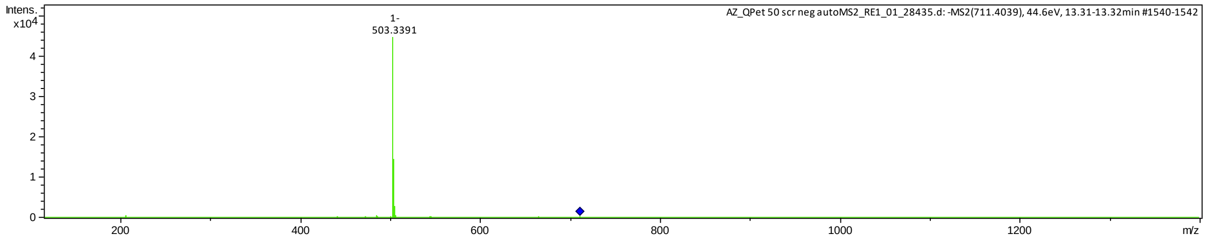

G 665/711, 13.31 min sericoside, arjunglucoside I, quadranoside III or isomer

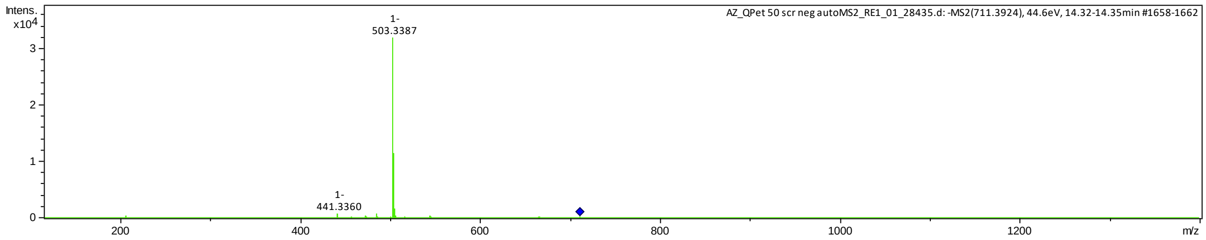

H 665/711, 14.34 min sericoside, arjunglucoside I, quadranoside III or isomer

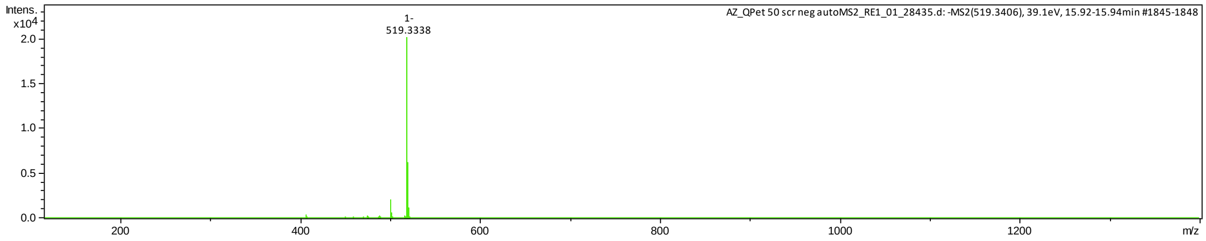

I 565/519, 15.92 min triterpene

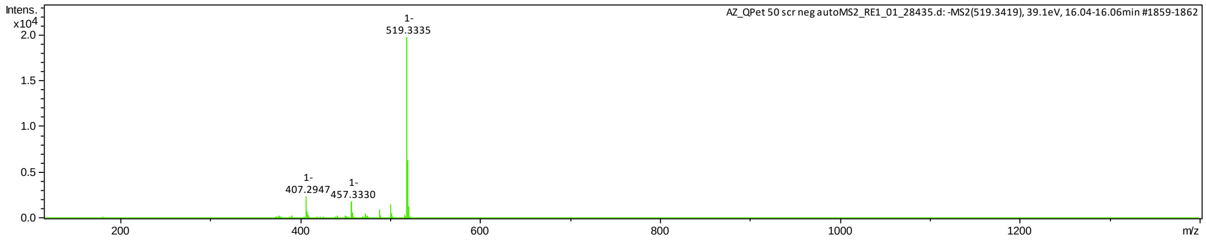

J 565/519, 16.04 min triterpene

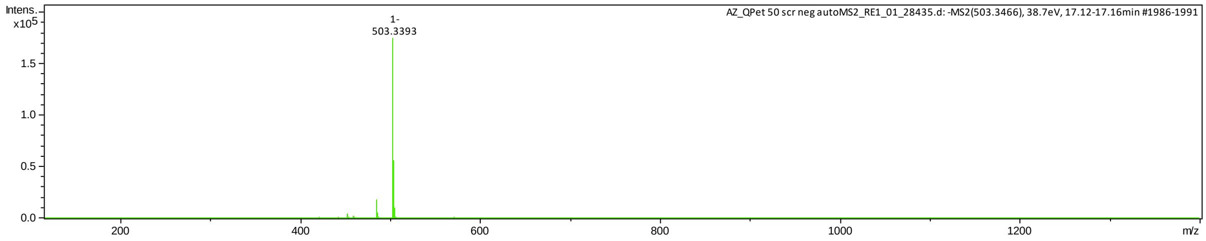

K 503/549, 17.15 min arjungenin or isomer

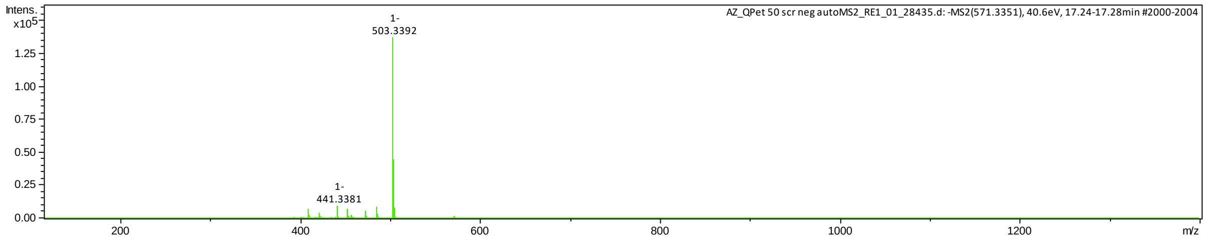

L 503/549, 17.25 min arjungenin or isomer

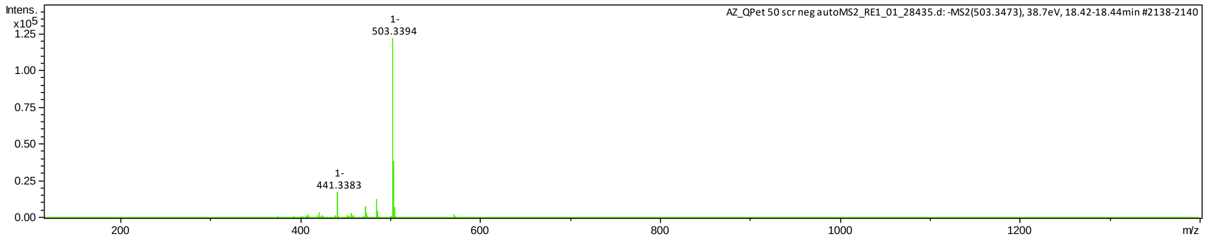

M 503/549, 18.41 min arjungenin or its isomer

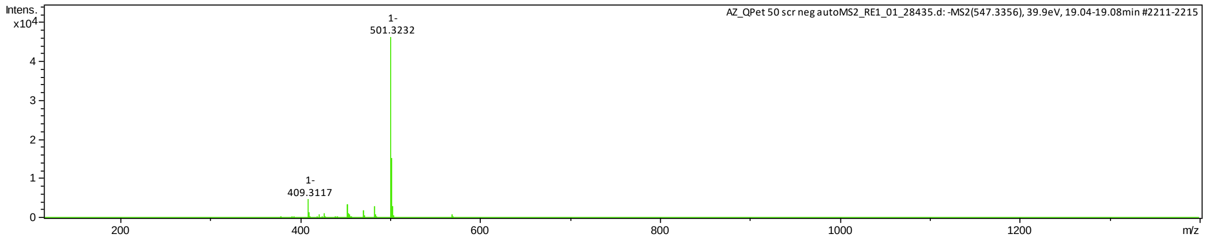

N 501/547, 19.06 min triterpene

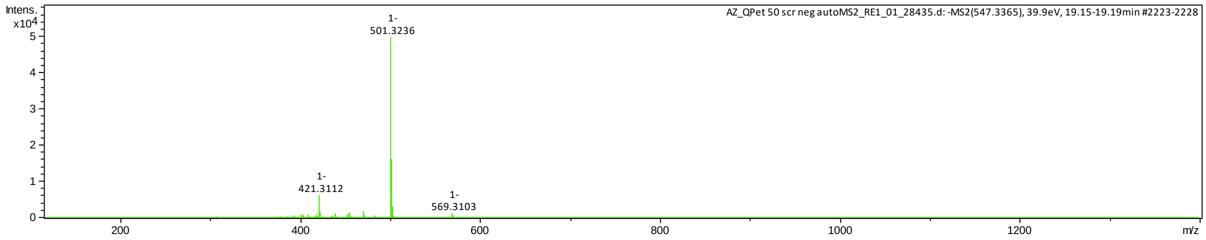

O 501/547, 19.16 min triterpene

Figure S3: A-O: MS2 spectra of distinctive constituents of analyzed *Quercus petraea*, *Quercus robur* and *Quercus rubra* acorn 70% aqueous methanol extracts.
